# Supplementary parenteral arginine corrects hypoargininaemia and rebalances plasma amino acid profiles in very preterm infants receiving parenteral nutrition: A prospective study

**DOI:** 10.1002/ncp.70077

**Published:** 2025-12-15

**Authors:** Frances Callaghan, Laura Burgess, Chandini Menon Premakumar, Diane McCarter, Eva Caamaño Gutièrrez, Daniel B. Hawcutt, Colin Morgan

**Affiliations:** ^1^ Neonatal Intensive Care Unit, Liverpool Women's Hospital Liverpool UK; ^2^ Department of Women's and Children's Health University of Liverpool Liverpool UK; ^3^ Faculty of Pharmacy, Universiti Kebangsaan Kuala Lumpur Malaysia; ^4^ Computational Biology Facility, Liverpool Shared Research Facilities, University of Liverpool Liverpool UK; ^5^ Institute of Systems, Molecular and Integrative Biology, University of Liverpool Liverpool UK; ^6^ NIHR Alder Hey Clinical Research Facility, Alder Hey Children's Hospital Liverpool UK

**Keywords:** administration, amino acids, critical care, immunonutrition, life cycle, neonates, nutrition, nutrition support practice, parenteral formulas/compounding, parenteral nutrition, research and diseases

## Abstract

**Background:**

Plasma amino acid (AA) profiles in parenteral nutrition (PN)–dependent very preterm infants (VPIs) consistently show overprovision of essential AA (EAA) and arginine deficiency. This may have implications for growth and immune/inflammatory responses. Aim: To compare plasma AA profiles on day 3 and day 10 in VPIs receiving standard PN (6.3 g/100 g AA arginine) and arginine‐supplemented PN (18 g/100 g AA) in VPIs at <30 weeks' gestation.

**Methods:**

VPIs were allocated (according to intervention PN availability) in a series of separate physiological studies to receive standard PN or arginine‐supplemented PN. This approach led to a final PN AA formulation design containing 18 g/100 g AA. Clinical, nutrition intake, and biochemical data were collected. Point‐of‐care testing was used to measure ammonia levels. Plasma AA levels were measured on days 3, 10 and 30 using ion exchange chromatography.

**Results:**

The highest mean daily arginine intake was on day 7: 521 mg/kg/day (142 mg/kg/day) at a dose of 18 g arginine/100 g parenteral AA. The median day 10 plasma arginine level was 85 (52–146) vs 41 (28–54) µmol/L for 18 g/100 g AA arginine vs control, respectively (*P* < 0.0001) The equivalent data for total EAA were 896 (750–1142) vs 1220 (1031–1428) µmol/L (*P* < 0.05) and blood ammonia levels were 46 (24–65) vs 51 (40–62) µmol/L (*P* = 0.28).

**Conclusion:**

In VPIs, PN arginine supplementation of 18 g/100 g AA increases arginine concentrations and reduces provision of EAA as demonstrated in the plasma AA profile. Higher plasma arginine levels are not sustained once parenteral arginine is discontinued. Blood ammonia levels were not useful in identifying individual arginine deficiency.

## Graphical abstract

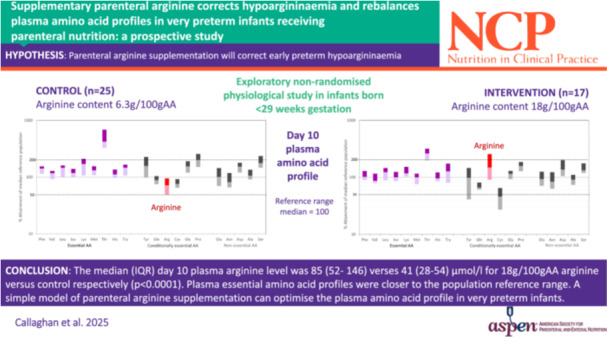

## INTRODUCTION

Since the original neonatal parenteral nutrition (PN) amino acid (AA) formulations were developed,^1^ there has been very little change in the relative proportions of individual AAs. This is despite a longstanding body of evidence that the PN‐dependent VPIs have suboptimal plasma AA profiles.^2^ These consistently show plasma levels of the nine essential AAs^3^, ^4^, ^5^ above those described for healthy preterm infants. Conversely, some conditionally essential Aas, including arginine, have plasma levels at the low end or below the population reference ranges. Some conditionally essential AAs (cysteine, tyrosine and glutamine) are unstable in PN formulations (so limiting PN AA content), but this is not the case for arginine.^6^ Cysteine supplementation of neonatal PN solutions particularly low in cysteine is recommended,^7^ but curiously, no equivalent recommendation applies to arginine. Systematic reviews have failed to demonstrate either clinically important benefits or harm when assessing evidence for early vs late^8^ or high‐ vs low‐dose^9^ parenteral AAs in PN‐dependent very preterm infants (VPIs) after birth. One potential explanation for this (other than heterogeneity of design and outcome measures) is that failings in the supply of individual AAs (too much or too little) may undermine attempts to improve outcomes by manipulating the total AA dose and/or timing. Thus, rebalancing the PN AA formulation could be an important design modification for future randomized controlled trials in this population.^10^

Arginine deficiency states readily occur during certain periods of growth and metabolism, notably in very preterm infants (VPIs).^9^ Intestinal metabolism of arginine precursors during enteral nutrition is essential to meet neonatal arginine requirements in animal^11^, ^12^ and human studies.^13^, ^14^ This makes the PN‐dependent preterm infant especially vulnerable to arginine deficiency. Hypoargininaemia in PN‐dependent neonates leading to hyperammonaemia is well described^15^ and reflects the important role arginine has in the hepatic urea cycle.^8^ Arginine plays a central role in several other metabolic pathways,^8^ including nitric oxide (NO) synthesis and polyamine and proline metabolism. The arginine‐NO pathway is an important mechanism by which hypoargininaemia may contribute to neonatal morbidity, particularly necrotizing enterocolitis (NEC) and potentially sepsis, pulmonary disease, and postoperative recovery.^16^ The Global Arginine Bioavailability Ratio (GABR) (arginine/[ornithine+citrulline]) has been proposed as a measure of NO synthesizing capacity^17^ in adults. The GABR has been associated with poorer outcomes in adult critical care,^17^ but GABR has not been reported in neonates. Arginine is a potent insulin secretagogue and deficiency is associated with preterm hyperglycaemia.^18^ Arginine comprises 14% protein AA in piglets,^19^ so as one of the key proteomic AA, arginine requirements of rapid preterm growth (with high rates of protein synthesis and turnover) may be particularly high.^11^ In arginine‐deficient animal models, early postnatal arginine supplementation improves neonatal growth.^20^

However, it is the potential for arginine to impact neonatal immune function^21^ and NEC that offers the greatest potential benefits to VPIs. Hypoargininaemia is associated with sepsis^16^, ^21^ and NEC.^22^ Mean plasma arginine levels of 41 µmol/L were associated with NEC,^22^ although there is uncertainty about normal reference range, with levels of 53–95 reported.^23^ The risk of NEC is reduced by both parenteral^23^, ^24^ and oral^25^, ^26^, ^27^ supplementation. The mechanism is not fully established but is likely to involve NO‐mediated changes in the gut microcirculation,^28^, ^29^ possibly with additional effects on lymphocyte function.^21^, ^30^, ^31^ Parenteral arginine supplementation has been recommended in recent international neonatal PN guidelines,^32^ but no dosage regimen or methodology is described.

The period of PN dependency in VPI coincides with a key period of immune system development and metabolic adaptation. To investigate the potential effects of increased parenteral arginine supplementation, we designed a sequence of physiological studies comparing a current licenced PN AA formulation (control) with the same formulation supplemented with arginine HCl. The target arginine content was guided by the doses used and plasma levels achieved in the studies showing a reduced risk of NEC. The final arginine‐supplemented PN AA formulation contained 18 g/100 g AA arginine. The overall AA dose remained unchanged.

The aims of this study series were to:

1. Describe the relationship between variations in parenteral arginine content (18 g/100 g AA) in the modified PN formulation and plasma arginine levels (and associated metabolites).
2. Describe the impact of parenteral arginine supplementation on the whole plasma AA profile with specific reference to total EAAs.

## METHODS

We undertook a series of ethically and regulatory‐approved exploratory studies conducted according to the guidelines of the Declaration of Helsinki. The Preterm Arginine INTake (PAINT) studies investigated biological pathways affecting immune function in infants requiring early PN (NCT02751437). The Research Ethics Committee (REC) and UK Integrated Research Application System (IRAS) details are: PAINT study: IRAS ID: 194333, Liverpool East REC 16/NW/0271 Approval date: 01.06.16. All serious unexpected adverse events and deaths were reported to and reviewed by the sponsoring organization in compliance with UK regulatory approvals. The studies used an iterative process using data from our systematic review^33^ to guide the choice of parenteral arginine supplementation. The protocol compared parenteral arginine supplementation of 6.3 g/100 g AA in control infants and an increasing parenteral arginine supplementation in the intervention group. A final PN formulation was modified to give 18 g/100 g AA arginine as the intervention (PAINT18). This final study received separate ethical and regulatory approval (NCT05299112) IRAS ID: 253730, HCRW (Wales) REC 20/NW/0044 Approval date: 03.06.20. The biochemical data were a planned secondary analysis.

Eligible infants were born <29 weeks' gestation and/or weighed <1200 g and were admitted to the neonatal intensive care unit at Liverpool Women's Hospital within 48 h of birth between September 2016 and December 2018 (PAINT) and again between December 2021 and March 2023 (PAINT18). Written parental consent was obtained within 72 h of birth. These single‐center physiological studies were unblinded and nonrandom, allocated to standard treatment or (according to stock availability) arginine‐supplemented PN. The control PN regimen remained unchanged throughout the whole study period and was that used in clinical practice. Control PN regimen was continued following parental consent whenever insufficient intervention group PN stock was available. This ensured control and intervention groups were recruited contemporaneously. Both control and intervention regimens were designed to give up to 3.8 g/kg/d protein (4.3 g/kg/d AA) and 105 kcal/kg/d energy and have been described previously.^34^ The AA dose was incrementally introduced over the first 5 days of life starting at 1.9 g/kg/d at birth.

All study infants received the control PN regimen as soon after birth as possible. All control infants continued to receive the unsupplemented parenteral regimen for the remainder of the study period. This parenteral AA has been described in detail previously^5^, ^34^ and used Vaminolact (Fresenius‐Kabi) as the parenteral AA source. This provided 6.3 g/100 g AA arginine to all control infants (*n* = 25). In the final study (PAINT18), in the intervention group (*n* = 17), arginine was added to the aqueous PN bag to achieve an arginine content of 18 g/100 g AA. The PN AA content of the control group (Vaminolact), final formulation (PAINT18 intervention), and the highest arginine content currently available in neonatal PN (Trophamine) are compared in Table 1. There were no differences in the micronutrients, vitamins, or electrolytes provided by the control or arginine‐supplemented regimens.

**Table 1:** Comparison of amino acid (AA) content (grams per 100 g AA) of human milk protein and manufacturers published data for three commonly used parenteral AA formulations: AA‐V (Vanimolact, Fresenius‐Kabi), AA‐V Arg 18 g/100 g AA, and AA‐T (Trophamine, Braun).

AA	Human milk^35^	AA‐V	AA‐T	AA‐V Arg 18 g/100 g AA
Phenylalanine (Phe)	3.7	4.1	4.8	3.6
Valine (Val)	5.4	5.5	7.8	4.8
Leucine (Leu)	9.9	10.7	14.0	9.4
Isoleucine (Iso)	5.5	4.7	8.2	4.1
Lysine (Lys)	6.9	8.6	12.0	7.5
Methionine (Met)	1.4	2.0	3.4	1.8
Threonine (Thr)	4.5	5.5	4.2	4.8
Histidine (His)	2.2	3.2	4.8	2.8
Tryptophan (Try)	1.7	2.1	2.0	1.8
Tyrosine (Tyr)	4.0	0.8	2.4	0.7
Glutamine (Gln)	0.0	0.0	0.0	0.0
Arginine (Arg)	3.9	6.3	12.0	18.0
Cystine (Cys)	2.1	1.5	0.4	1.3
Glycine (Gly)	2.3	3.2	3.6	2.8
Proline (Pro)	8.2	8.6	4.1	7.5
Glutamate (Glu)	17.2	10.9	5.0	9.5
Aspartate (Asn)	0.0	0.0	0.0	0.0
Asparagine (Asp)	8.9	6.3	3.2	5.5
Alanine (Ala)	3.8	9.6	5.4	8.4
Serine (Ser)	4.7	5.8	3.8	5.1
Ornithine (Orn)	0.0	0.0	0.0	0.0
Taurine (Tau)	0.0	0.5	0.3	0.4

All infants received clinical care in accordance with Liverpool Women's Hospital PN protocols, including fluid management; introducing, increasing, and stopping enteral feeds; and biochemical monitoring. The study intervention continued until 14 completed days of life. PN was discontinued once enteral feeds exceeded 75% total fluid volume. The transition from PN to enteral feeds involved the preferential use of expressed or donor breast milk, which remained unfortified until 150 ml/kg/day enteral feeds.^34^ Patient data were collected from the electronic patient record. Daily enteral/parenteral protein/energy intake data were calculated as described previously.^5^, ^34^ Individual AA intake was calculated from manufacturers' data for composition of PN or formula and estimated from published average values for human milk proteins.^35^ The AA intake calculations from human milk take into account the proportion of individual AAs remain the same although all individual AAs increase as a result of increased protein content in preterm vss term mothers' own milk.^36^, ^37^ Average protein intakes for AA intakes were adjusted for preterm colostrum, transition and milk from mothers' own milk, and mature term milk data for donor breast milk feeds.^35^, ^36^, ^37^

The plasma AA profile was obtained on day 3 (where the clinical protocol increases the AA intake to 2.9 g/kg/day). In the intervention (18 g/100 g AA) group, sampling occurred before the intervention was started. A further plasma AA sample was taken on day 10 in all infants, as described in our previous work.^5^ AAs were measured using ion exchange chromatography with normal reference ranges obtained from a recent multicenter UK study of infants aged <6 months (including our own laboratory) and using the same analysis technique.^38^

Blood ammonia levels (µmol/L) were measured using a point‐of‐care (POC) testing device at approximately the same time when the plasma AA blood samples were taken. The PocketChem BA analyzer was used together with the Arkray Ammonia Test Kit II reagent strips. The 0.02 ml capillary blood sample was processed within 30 min from time of collection. Blood ammonia levels of >100 µmol/L required repeat testing, and if repeat test confirmed levels were above the limit, laboratory verification from the Alder Hey Children's Hospital Pathology Laboratory was needed. Arterial or capillary blood gas samples were performed with a frequency indicated by local policy and clinical needs of the infant and provided blood glucose and lactate measurements. These data were collected during the PAINT18 study, which allowed the mean daily blood lactate, base deficit, and glucose levels to be estimated based on the number and timing of samples during each 24‐h period. For example: 4 samples in a 24‐h period contribute proportionately to the crude estimated mean based on the timing of each sample, as described previously.^19^ The clinical monitoring of glucose/lactate after day 14 becomes much less frequent as the transition from parenteral to enteral feeds is completed in most infants by then. All routine biochemical, hematological, and microbiological data were collected from the electronic data management system. Similarly, records were obtained for any drug treatments needed to support circulation (inotrope infusions or hydrocortisone) or manage blood glucose control (insulin) during the intervention period.

### Statistical analyses

The data compare all controls combined (arginine content 6.3 g/100 g AA) and PAINT18 intervention (arginine content 18 g/100 g AA). Data S1 is provided for the intervention groups that received 12–15 g/100 AA. For normally distributed data (primarily nutrient intake and biochemical monitoring), *t* tests were used for continuous variables and Fisher exact test analyses for categorical variables. Statistical analyses for nonnormally distributed data were undertaken with Mann‐Whitney tests.

Most data (including all the main outcomes) did not follow a normal distribution and have been presented as median (interquartile range [IQR]). Statistical analyses were undertaken in the software R.^39^ Day 10 AA differences between all levels of intervention were evaluated with a Kruskal‐Wallis test, followed by a Conover Iman post hoc test (package conover.test^40^) to identify pairwise comparisons. All *P* values were adjusted for false discovery rate using Benjamini‐Hochberg method. These statistical analyses, some extra visualizations, and the data are publicly available in GitHub repository https://github.com/EvaCaamano/PAINT_AminoAcidStudies.

## RESULTS

The demographic data of all controls (*n* = 25) and high (arginine content 18 g/100 g AA) intervention (*n* = 17) groups are summarized in Table 2 along with nutrition intakes. These data show statistically significantly lower intake of parenteral EAAs and higher intake of arginine in the intervention group. The key plasma data are shown in Table 3. The median (IQR) day 10 plasma arginine level was 85 (52–146) vs 41 (28–54) µmol/L for high (18 g/100 g AA) arginine vs control, respectively (*P* < 0.0001). The median (IQR) day 10 plasma EAAs were 896 (750–1142) vs 1220 (1031–1428) µmol/L (*P* < 0.05). The median day 10 blood ammonia was 46 (24–65) vs 51 (40–62) µmol/L (*P* = 0.28) in the control and intervention groups, respectively. Plasma levels of arginine metabolism intermediates (not present in PN AA formulation) showed statistically significantly higher levels of ornithine and citrulline with arginine supplementation but no differences in glutamine (Table 3). The mean (SD) GABR was 0.43 (0.11) and 0.32 (0.2) in the intervention and control groups, respectively (not statistically significant). If infants not receiving any parenteral arginine on day 10 are excluded from these data, then median (IQR) plasma arginine levels are 118 (82–156) with 18 g/100 g AA (*n* = 12) vs 41 (25–54) in controls (*n* = 22) in the respective subgroups (*P* < 0.001). The corresponding data for EAA are 1032 (845–1205) and 1257 (965–1623) for intervention and control, respectively (not statistically significant).

**Table 2:** Comparison of d10 survivors' basic demographics at birth and nutritional intake data: mean (sd) daily intakes (day1‐10) of total and parenteral intakes of protein(g/kg/d), essential amino acids (EAA) and arginine in arginine 18 g/100 g AA (high) group and all control groups combined (adjusted p‐values shown).

	Arg 18 g/100 g AA (*n* = 17)	Control (*n* = 25)	Padj
Sex (M:F)	12:5	15:10	**‐**
Gestation	26.4 (1.8)	26.8 (2.3)	**0.55**
Birthweight	987 (239)	883 (201	**0.14**
Total protein intake	2.97 (0.27)	3.04 (0.33)	**0.79**
Parenteral protein intake	2.19 (0.43)	2.60 (0.36)	**0.59**
Total EAA intake	1317 (118)	1533 (161)	**<0.001**
Parenteral EAA intake	987 (195)	1350 (188)	**<0.001**
Total Arginine intake	382 (75)	206 (18)	**<0.001**
Parenteral arginine intake	350 (88)	185 (26)	**<0.001**
Max parenteral arginine	602 (92)	245 (25)	**<0.001**

**Table 3:** Comparison of survivors' day 10 median (IQR) plasma levels of the 22 reported AAs (µmol/l) between arginine 18 g/100 g AA (high) group and all control groups combined (adjusted *P* values shown).

Amino acid	Arginine 18 g/100 g AA (*n* = 17)	Control (*n* = 25)	Padj
Phenylalanine (Phe)	53 (47–67)	74 (60–81)	<0.001
Valine (Val)	126 (115–168)	172 (135–190)	0.004
Leucine (Leu)	119 (86–141)	142 (98–157)	0.022
Isoleucine (Iso)	50 (43–62)	59 (50–70)	NS
Lysine (Lys)	180 (159–236)	265 (166–325)	NS
Methionine (Met)	25 (19–29)	33 (27–39)	NS
Threonine (Thr)	254 (190–309)	409 (319–665)	NS
Histidine (His)	71 (63–90)	83 (72–101)	NS
Tryptophan (Try)	19 (12–26)	22 (16–25)	NS
Tyrosine (Tyr)	57 (24–85)	92 (57–132)	NS
Glutamine (Gln)	370 (342–452)	498 (418–590)	NS
Arginine (Arg)	85 (52–146)	41 (28–54)	<0.001
Cystine (Cys)	17 (10–26)	28 (24–34)	NS
Glycine (Gly)	284 (229–321)	383 (296–469)	0.029
Proline (Pro)	245 (197–285)	312 (233–396)	0.010
Glutamate (Glu)	98 (71–125)	101 (70–145)	NS
Asparagine (Asn)	32 (24–47)	31 (25–45)	<0.001
Aspartate (Asp)	26 (20–38)	29 (23–34)	<0.001
Alanine (Ala)	271 (221–327)	317 (273–405)	NS
Serine (Ser)	169 (151–223)	224 (187–300)	0.087
Ornithine (Orn)	249 (82–396)	112 (79–163)	<0.001
Citrulline (Cit)	11 (8–12)	13 (10–17)	0.015

*Note*: NS = not significant after Kruskal‐Wallis test.

**Figure 1:**
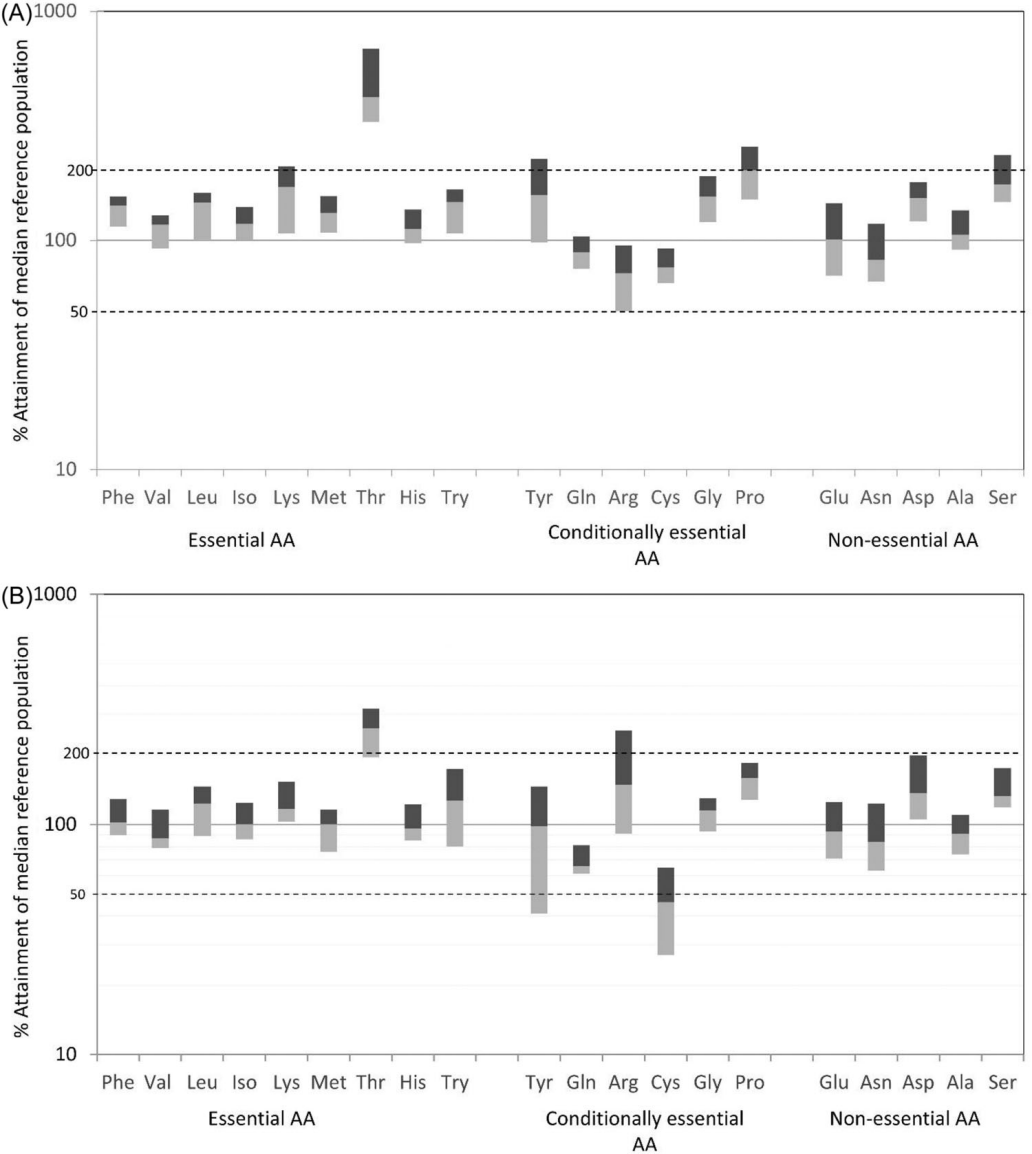
A and 1B. Compares the plasma amino acid (AA) data in the (A) combined control group and (B) arginine (18 g/100 g AA intervention group) using the same reference population.^32^ The individual median plasma AA levels and interquartile range have been converted to percentages of the corresponding reference plasma AA median (reference median = 100). For definitions of AA abbreviations, see Table 1.

Table 3 also summarizes the remaining proteomic AAs, showing median (IQR) individual plasma AA levels on day 10 for the control and intervention groups. As well as arginine, there were significant differences in phenylalanine, cysteine, glycine, asparagine and aspartamine. In addition, the 20 proteomic AA median (IQR) values have been converted to percentages of the reference population^32^ medians in Figure 1A and 1B, as previously described,^5^ to demonstrate how both hypoargininaemia and the plasma levels of the nine individual EAAs benefit from the arginine‐supplemented formulation used in the PAINT18 intervention group. Of the latter, seven median EAA plasma levels remain at or above the reference range medians, with two just below (Figure 1B).

**Figure 2:**
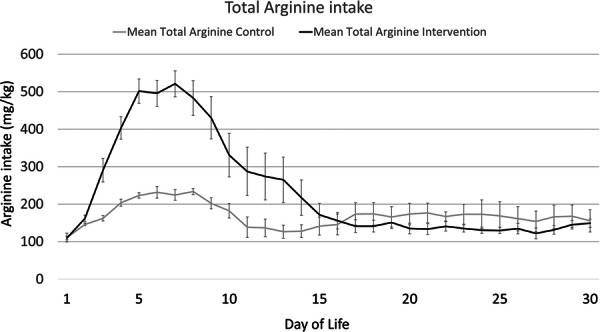
Mean (SEM) daily total (enteral and parenteral) arginine (mg/kg/d) intake in PAINT18 intervention (arginine 18 g/100 g AA) and PAINT18 controls over the 30 day study period.

The daily parenteral arginine intake in each of the first 30 days of life (PAINT18 infants only) is shown in Figure 2. Parenteral arginine intake peaks on day 7 in the PAINT18 intervention group and falls significantly as infants transition to milk feeds. By day 30, only three infants (two controls) were PN‐dependent. Median (IQR) plasma arginine levels were 80 (38–88) mmol/L and 56 (43–79) mmol/L and median (IQR) blood ammonia levels were 63 (39–78) and 64 (55–77) in the control (*n* = 6) and intervention (*n* = 13) groups, respectively (not statistically significant).

There were three deaths in the first 14 days of life, all in the intervention group. One survived long enough to have a sample on day 10 and is included in the survivors' data. The three infants were 23.4, 23.7, and 24.6 weeks' gestation and with birthweight 665, 670, and 715 g respectively. One deteriorated between consent and intervention starting (and died 12 h later). Mortality in the 30‐day study period is shown in Table 4. Details of circulatory support (inotropes and/or hydrocortisone) and infection (positive blood cultures and *C*‐reactive protein [CRP]) are also provided in Table 4. These data indicate that the infants receiving the intervention had more of these complications before the study PN started than control infants although overall there were no statistically significant differences. CRP was raised for a total of 9 days (7.5% of all days in the study intervention period) in all control group infants and 8 days (3.9%) in the intervention group. No babies developed NEC, but two infants had spontaneous intestinal perforation: 1 in each group.

**Table 4:** PAINT18 infants demographic data with comparison of mortality (up to day 30) and numbers of infants receiving circulatory support, with a positive blood culture and raised inflammatory markers (*C*‐reactive protein > 20 mmol/L) up to the end of the intervention period (day 14) during the intervention period (in all infants recruited to PAINT18 control (*n* = 9) and intervention (*n* = 19) groups.

	Arg 18 g/100 g AA (*n* = 19)	Control (*n* = 9)	*P*
**Day 1–30 (study period)**			
Sex (male to female)	13:6	5:4	**0.68**
Gestation (weeks)	26.1 (1.8)	26.3 (1.5)	**0.63**
Gestation of <25 weeks, (*n*) (deaths before day 30)	5 (4)	2 (1)	**0.63** *
Gestation of >25 weeks, (*n*) (deaths before day 30)	14 (1)	7 (0)	
Birthweight	956 (239)	909 (167)	**0.67**
**Day 1**–**14 (intervention period)**			
Circulatory support (%)	7/19 (37%)	2/9 (22%)	**0.67**
Prestudy PN	4/7 (56%)	0/2	
Blood culture positive	7/19 (37%)	3/9 (33%)	**1.0**
Prestudy PN (%)	2/7 (28%)	0/3	
CRP >20mmol/L	4 (21%)	2 (22%)	**1.0**
Prestudy PN	3/4 (75%)	1/2 (50%)	

*Note*: Of those infants receiving circulatory support, positive blood culture or raised inflammatory markers, the proportion of infants receiving circulatory support, positive blood culture or raised inflammatory markers before the study PN started (either intervention or control) is also shown. In the 19 intervention patients, 7 needed circulatory support however in 4/7 infants needing circulatory support, the support started in the time period BEFORE the intervention started (called prestudy‐PN in the table). The infants are actually receiving control PN before the study period starts and switch to intervention PN. In the control group 2/9 infants needed circulatory support but none (0/2) were in the prestudy PN period (these infants remain on the control PN after the study period starts). This is included in the data as it adds significantly to the interpretation of the findings and underpins corresponding points in the results and discussion.

Abbreviations: CRP, C‐reactive protein; PN, parenteral nutrition.

* all deaths combined.

No difference in glucose or lactate levels were identified throughout the intervention period (Figure 3A and 3B). Figure 3C and 3D summarizes the mean daily blood glucose, base deficit, lactate, and chloride levels (mmol/L) for the intervention period (days 1‐15). Both groups had hyperchloremia in the first 24 h after birth: mean (SD) plasma chloride levels were 110 (2.2) vs 114 (2.5) in the control and intervention groups (*P* < 0.01). The maximum chloride levels occurred on day 3: 111 (4.8) vs 117 (5.4) (*P* = 0.021) on the day the intervention started but then fell thereafter in both groups (Figure 3D). The mean daily chloride levels over the intervention period were 111.3 (4.2) and 107.5 (5.2) mmol/L in intervention and control groups, respectively (*P* = 0.054). This is likely to have contributed to greater base deficits seen between the groups. The mean daily base deficit over the intervention period was −5.0 (2.4) and −2.8 (1.5) in the intervention and control groups, respectively (*P* = 0.02).

Figure 3Mean (SEM) daily blood glucose (A), lactate (B), base excess (C) and plasma chloride (D) mmol/L in PAINT18 intervention (18 g/100 g AA) and PAINT18 control infants over the first 15 days of life.

**Figure:**
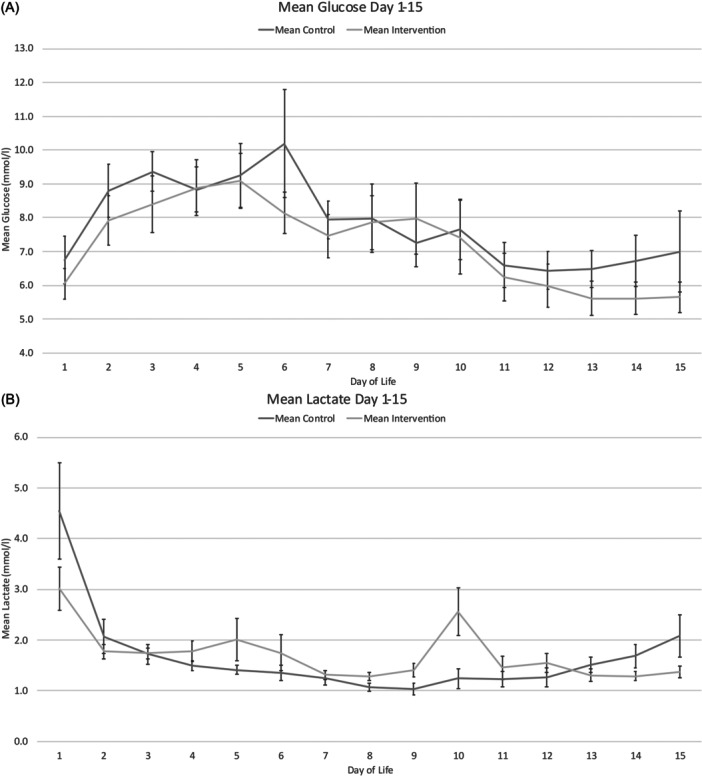

**Figure:**
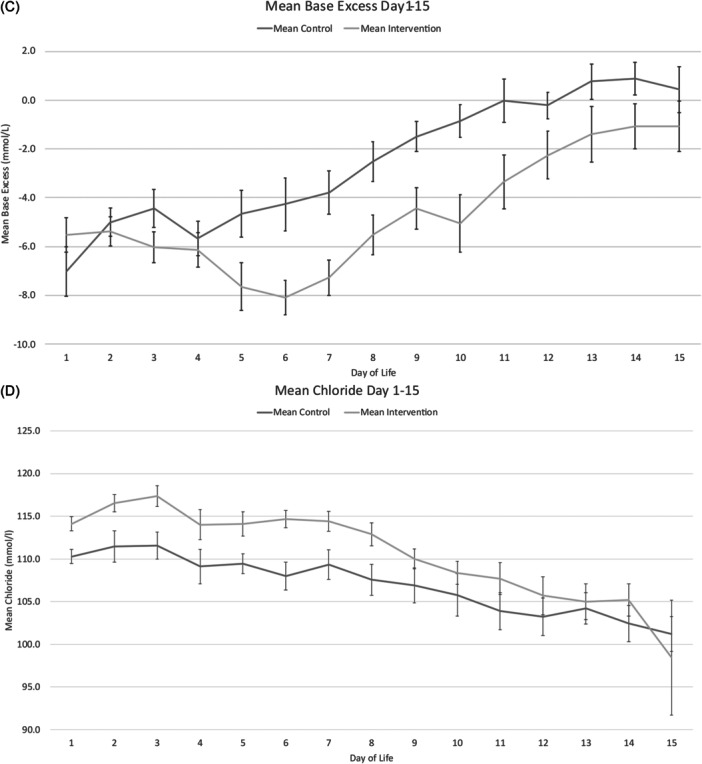

## DISCUSSION

This physiological study describes the effect of parenteral arginine supplementation (18 g/100 g AA) on the plasma AA profile of VPIs. The formulation was developed as part of an iterative process and supplementary data from the intermediate dose formulations are available. The supplementation not only corrects hypoargininaemia but also reduces the plasma levels of many individual EAAs by adjusting the relative proportion of EAAs in the modified PN AA formulation. This brings them closer to the published reference ranges. This study also shows efficacious dosing of parental arginine can be achieved using existing commercial PN formulations allowing the recent European guidance on arginine supplementation^32^ to be incorporated safely and rapidly into current neonatal clinical practice.

The day 10 plasma data (arginine content 18 g/100 g AA) indicate that plasma arginine levels fall back to baseline as the infants transitions to full enteral feeds. This is confirmed by the day 30 data when nearly all infants were enterally fed. This is of clinical importance because the median postnatal age of onset for NEC was reported as day 17 (IQR: 12–27) for both medically and surgically treated presentations in a UK population^41^ and systematic review.^42^ The variation in the day 10 plasma arginine was affected by the proportion of PN feeding at the time. This is consistent with our previous systematic review of the relationship between parenteral arginine intake and plasma arginine levels.^33^ This implies that the peak arginine level is likely to occur earlier on day 7 to coincide with peak arginine intake. Amin et al.^24^ reported higher plasma arginine levels (159 µmol/L on day 14) when supplementing with parenteral arginine supplementation to achieve a similar content as in PAINT18 infants (18 g/100 g AA). However, plasma arginine levels were sustained by supplementing infants with oral arginine after PN finished. Our study was designed to investigate the impact of parenteral arginine supplementation only. This clearly indicates that if raised plasma arginine levels need to be sustained until day 30 to protect infants from NEC, then oral therapy is required. Nevertheless, a peak plasma arginine on day 7 may still have therapeutic importance given hypoargininemia on day 7 is associated with later development of NEC.^43^ Oral arginine therapy may not be well absorbed or tolerated (if no milk feeding is possible or there are bile‐stained aspirates) at this postnatal age, meaning plasma levels will be entirely dependent on parenteral arginine provision. Despite encouraging pharmacokinetic data in animals,^44^ more understanding is required of the interplay between parenteral and enteral arginine supplement in this complex heterogeneous preterm population.

These data show arginine supplementation may impact other key intermediates, such as ornithine, citrulline and glutamine. Notably, none of these AAs are present in the PN AA formulation, meaning that changes in plasma levels reflect changes in metabolism not intake. The GABR has been proposed as a measure of NO synthesizing capacity, with lower GABR associated with a range of adult morbidities.^17^, ^45^ It is not known whether the GABR is of value in predicting preterm outcomes, but our data show any potential increase in GABR (due to increased plasma arginine levels) was offset by increased plasma ornithine levels after arginine supplementation. More recent evidence suggests other arginine metabolites (asymmetric dimethylarginine [ADMA]) have replaced GABR as a biomarker for adverse outcomes in an adult intensive care setting,^46^ but unlike the AA intermediates above, ADMA is not part of a clinical neonatal AA profile.

Arginine metabolic pathways are closely involved in the urea cycle and ammonia metabolism. Increasing plasma arginine levels was not associated with a reduction in plasma ammonia levels as reported elsewhere,^22^, ^24^ except on day 3 of life. This is consistent with recently published data on preterm plasma ammonia levels in the first week of life.^47^ POC ammonia measurement potentially offered a rapid, low‐volume sample method to monitor functional arginine deficiency in individual patients, but our data suggest it has little value in monitoring hypoargininemia associated with nutrition deficiency. There were no adverse effects on glucose metabolism noted. VPIs are at high risk of hyperglycemia in the first 2 weeks of life and, therefore, arginine may have potential benefits as an insulin secretagogue during this period as described in a previous study.^18^ The intervention group did show evidence of a metabolic disturbance in acid base balance. However, the hyperchloremic acidosis in the arginine supplementation group arose from non‐PN sources of chloride that increased plasma chloride levels before the intervention started. The study PN formulation was designed to ensure total PN chloride intake was not increased, and this approach appears evident in the falling plasma chloride after arginine‐supplemented PN was started.

Correcting the overprovision of EAAs could be as important as preventing hypoargininemia given the association between AA dose and sepsis shown in both term^48^ and preterm neonates.^49^ Unfortunately, none of this work investigated plasma AA profiles. Despite the reduction in EAA intake, none of the nine individual EAAs demonstrated median plasma levels that were <90% of the original reference range median (7 remained >100% of the reference range median). This indicates that the rebalancing has not led to any individual EAA deficiency. One disadvantage of rebalancing parenteral AA intake using only arginine is that deficiency of other conditionally EAA (in which PN concentration is limited by solubility and stability) may be aggravated. Our data suggest this may be a risk for glutamine and cysteine. Supplementation regimens have been described for cysteine^50^ and glutamine^51^ although they have not been associated with clinical benefits.^52^, ^53^ Given that glutamine is an intermediate in arginine metabolism, this needs further investigation.

There are limits in using individual plasma AA levels to interpret individual AA status. However, there are no other realistic alternatives in this clinical population (and indeed plasma AA profiles are rarely part of routine PN monitoring). Moreover, given the association reported between hypoarginemia and NEC^22^ and then arginine supplementation and prevention of NEC,^24^ we believe it is reasonable to suggest plasma arginine levels as a target to guide therapeutic intervention. The literature does not provide clear guidance as to a minimum arginine threshold, and the value of our study is that it describes for the first time what is achievable in clinical practice during the early postnatal period. The change in arginine exposure between control and PAINT18 groups increases nearly threefold from 6.3 to 18 g/100 g AA. In contrast, the reduction of all other individual Aas, including the nine EAAs, is ≤10%. It is possible that with such a large change in plasma arginine, bioavailability of other AAs could be affected through competition for transmembrane transport mechanisms, and this may be more important than the provision of individual parenteral AAs per se. However, interpreting the metabolic interactions and impact for individual AAs following such small changes is beyond the scope of this article.

Hypoargininemia is associated with NEC, but the implied target plasma arginine level (from Amin et al.^24^) required to prevent NEC (mean >150 µmol/L) may be much higher than that of reference population data (mean 57 µmol/L). The current evidence does not provide further clarification as to where the minimum target plasma arginine level lies in this range. Our data indicate how difficult it is to achieve and sustain a rise in plasma arginine levels using only parenteral arginine supplementation. Our data also showed hypocitrullinemia. Low enterocyte capacity to synthesize citrulline during periods of PN dependency and low enteral feed intakes is well recognized. Citrulline is the precursor to arginine. The association between low citrulline levels and NEC is well recognized in human and animal studies. The proposed mechanism by which high plasma arginine may reduce the risk of NEC involves NO.^24^ Arginine is the sole substrate for NO^17^ and involves synthesis by three different NO synthase (NOS) isoenzymes^54^ that are associated with inflammation and NEC in human infants.^55^, ^56^ NEC pathogenesis involves links between NO (given its role in endothelial relaxation), inhibition of platelet aggregation, and modulation of inflammation.^57^ However, an important theoretical risk from plasma arginine levels well above the reference range is excess NO production, particularly during sepsis. In animal and adult models, there is still controversy as to whether arginine supplementation during acute sepsis is harmful or beneficial.^17^ This study is not large enough to provide reassurance on this question but highlights that this is a population at high risk of mortality and infection. The three deaths during arginine supplementation were all born at <25 weeks' gestation and had other early risk factors (two had evidence of early‐onset sepsis) raising the risk of mortality further. The only previous study of parenteral arginine supplementation that achieved higher plasma arginine levels than our study reported three deaths (<25 weeks' gestation)^24^ in the intervention group and none in the controls. However, 23 (9.1%) eligible infants died in the first 5 days of life and a further 31 eligible infants were excluded (28 due to significant intraventricular hemorrhage). This indicates the study population was much lower risk of mortality than the infants included in our population. The study also reported no differences in sepsis or hypotension.^24^ We found no evidence of high lactate levels to suggest perfusion was compromised in our study. However, the interpretation is limited because the intervention group appeared sicker (more inotrope use to treat hypotension, infection, and raised inflammatory markers) before the intervention PN was started. The study is not powered to address these concerns fully, and plasma levels may not reflect activity in different body organs or the immune system.

The key strength of this study is that for the first time, the impact of parenteral arginine supplementation alone on plasma arginine levels in 2 weeks immediately after birth at <29 weeks has been described. The population studied is at high risk of NEC. NEC is the only complication for which there is evidence to support arginine supplementation despite other potential benefits.^16^ The approach described is readily translated into clinical practice. There appears to be individual variation as well as variation secondary to the speed of transition to enteral feeds. In addition, the control group combines infants recruited in three separate epochs over 5 years. There were no major PN policy changes during this time, but other confounding factors cannot be excluded. Moreover, the plasma AA profile for the combined control group closely resembles the plasma AA profile described for an intervention group receiving the same PN regimen in our previous study.^5^ Extensive physiological data were available from daily clinical monitoring, but this was reduced when enteral feeding was established. Thus, the data available after the period of PN dependency are more limited. Nevertheless, the detailed physiological data in the first 14 days of life provides much more detail than previously published during parenteral arginine supplementation.^16^, ^24^ There are some reassurances relating to blood glucose control and an indication of the impact of arginine HCl on chloride intake. Nevertheless, the nonrandomized small physiological design means that theoretical concerns about short‐ and long‐term clinical risks cannot be definitively addressed.

The fact that parenteral arginine alone cannot sustain plasma arginine levels associated with a reduced rate of NEC is an important clinical finding. The wider AA profile data helps to broaden the context beyond the primary aim of arginine supplementation by considering other AAs. Clearly, it is not possible to supplement parenteral arginine without reducing the proportion of other AAs. We have shown this has potential benefits for EAA provision in that the plasma profile of individual EAAs is closer to their respective reference ranges than before supplementation. The arginine supplementation model benefits from being simple, and although we believe the plasma AAs are closer to the reference ranges for most AAs, a more complex supplementation model involving other conditionally EAAs may achieve even more. Although an important aim was to correct hypoargininemia (as defined by the reference population data), plasma arginine levels change quickly in response to changes in parenteral intake, and fixing the postnatal day of measurement may not be representative of the peak level. This is supported by the higher plasma arginine levels in the subgroup of infants still receiving some parenteral arginine (18 g/100 g AA) on day 10. More frequent sampling (using residual blood volumes) using more targeted ELISA analysis of arginine and ADMA levels could be incorporated as a future methodology.^58^ This would provide more detail about longer‐term arginine supplementation strategies, as well as a potential method for risk stratifying individuals at higher risk of NEC or sepsis. Future arginine supplementation studies should prioritize oral arginine supplementation therapy and whether it alone is sufficient to sustain early plasma arginine levels associated with reduced NEC or whether a parenteral arginine source is still essential.^16^

## CONCLUSION

We conclude that arginine supplementation of existing PN AA formulations to achieve 18 g/100 g AA prevents hypoargininemia and reduces overprovision of EAAs. This results in a plasma AA profile that more closely resembles that of enterally fed preterm and term infants than seen with unsupplemented PN AA formulations. Plasma levels well above the reference range (and associated with a reduced risk of NEC) are achievable but not sustained outside the period of PN dependency. No adverse metabolic effects attributable to arginine supplementation were identified, but definitive investigation of the potential risks and benefits is still required.

## AUTHOR CONTRIBUTIONS

Frances Callaghan, Laura Burgess, Diane McCarter, Chandini Menon Premakumar were all involved in parts of the study design, recruitment, data collection and analysis; Eva Caamaño Gutièrrez provided statistical advice and performed the statistical analyses; Daniel B. Hawcutt supported funding applications and advised on design and manuscript draft preparation; and Colin Morgan supported all elements of the study from conception and design through to final analyses and is the corresponding author. All authors reviewed and approved manuscript drafts and approved the final version.

## CONFLICT OF INTEREST STATEMENT

Colin Morgan, Chandini Menon Premakumar, and Laura Burgess are named on a UK national patent filing concerns arginine supplementation of parenteral nutrition: NEONATAL PARENTERAL NUTRITION FORMULATIONS' PCT/GB2019/051206. Priority 01/05/2018. This did not proceed to National Filings and there have been no commercial or financial interests as a result. The remaining authors declare no conflict of interests.

## Supporting information

NCP 2024 rev2.
