# Supplementary material for: Supplementary parenteral arginine corrects hypoargininaemia and rebalances plasma amino acid profiles in very preterm infants receiving parenteral nutrition: A prospective study
Source: Nutr Clin Pract. 2025 Dec 15;41(2):619–32. doi: 10.1002/ncp.70077 (PMC12982625; doi:10.1002/ncp.70077)
Supplement: Supplementary file 1 — NCP 2024 rev2. [file NCP-41-619-s001.docx]

**Supplementary files**

**Supplementary Table S1: Comparison of basic demographics at birth and nutritional intake data: mean (sd) daily intakes (day1-10) of total and parenteral intakes of protein(g/kg/d), essential amino acids (EAA) and arginine in arginine 18g/100gAA (high) group and all control groups combined (adjusted p-values shown). Arginine 12-15g/100gAA (moderate) group data for descriptive purposes only but statistic results available in the supplementary information.**

|  | **Arg 18g/100gAA**  **(n=17)** | **Arg 12-15g/100gAA**  **(n=26)** | **Control**  **(n=25)** | **Padj** |
| --- | --- | --- | --- | --- |
| Sex (M:F)  Gestation  Birthweight | 12:5  26.4 (1.8)  987 (239) | 14:12  27.0 (2.3)  888 (231) | 15:10  26.8 (2.3)  883 (201 | **-**  **0.55**  **0.14** |
| Total protein intake  Parenteral protein intake  Total EAA intake  Parenteral EAA intake  Total Arginine intake  Parenteral arginine intake  Max parenteral arginine | 2.97 (0.27)  2.19 (0.43)  1317 (118)  987 (195)  382 (75)  350 (88)  602 (92) | 3.15 (0.25)  2.71 (0.29)  1478 (110)  1298 (141)  374 (48)  355 (50)  482 (62) | 3.04 (0.33)  2.60 (0.36)  1533 (161)  1350 (188)  206 (18)  185 (26)  245 (25) | **0.79**  **0.59**  **<0.001**  **<0.001**  **<0.001**  **<0.001**  **<0.001** |

**Supplementary Table S2: Comparison of day 10 median (IQR) plasma levels of the 22 reported AA (µmol/l) between arginine 18g/100gAA (high) group and all control groups combined (adjusted p-values shown). Arginine 12-15g/100gAA (moderate) group data for descriptive purposes only but statistic results available in the supplementary information. NS=not significant after Kruskal-Wallis test.**

| **Amino acid** | **Arginine 18g**  **per 100gAA (n=17)** | **Arginine 12-15g**  **per 100gAA (n=26)** | **Control**  **(n=25)** | **Padj** |
| --- | --- | --- | --- | --- |
| Phenylalanine (Phe)  Valine (Val)  Leucine (Leu)  Isoleucine (Iso)  Lysine (Lys)  Methionine (Met)  Threonine (Thr)  Histidine (His)  Tryptophan (Try)  Tyrosine (Tyr)  Glutamine (Gln)  Arginine (Arg)  Cystine (Cys)  Glycine (Gly)  Proline (Pro)  Glutamate (Glu)  Asparagine (Asn)  Aspartate (Asp)  Alanine (Ala)  Serine (Ser)  Ornithine (Orn)  Citrulline (Cit) | 53 (47-67)  126 (115-168)  119 (86-141)  50 (43-62)  180 (159-236)  25 (19-29)  254 (190-309)  71 (63-90)  19 (12-26)  57 (24-85)  370 (342-452)  85 (52-146)  17 (10-26)  284 (229-321)  245 (197-285)  98 (71-125)  32 (24-47)  26 (20-38)  271 (221-327)  169 (151-223)  249 (82-396)  11 (8-12) | 71 (68-119)  149 (134-171)  114 (105-131)  55 (46-60)  226 (186-301)  34 (25-39)  467 (327-595)  87 (83-102)  18 (14-26)  90 (68-119)  466 (389-577)  58 (38-75)  25 (22-34)  409 (358-479)  294 (243-327)  117 (100-142)  46 (33-55)  28 (23-34)  301 (279-359)  251 (215-289)  165 (113-227)  14 (13-17) | 74 (60-81)  172 (135-190)  142 (98-157)  59 (50-70)  265 (166-325)  33 (27-39)  409 (319-665)  83 (72-101)  22 (16-25)  92 (57-132)  498 (418-590)  41 (28-54)  28 (24-34)  383 (296-469)  312 (233-396)  101 (70-145)  31 (25-45)  29 (23-34)  317 (273-405)  224 (187-300)  112 (79-163)  13 (10-17) | 0.00001  0.00438  0.02189  NS  NS  NS  NS  NS  NS  NS  NS  <0.00001  NS  0.02911  0.01013  NS  0.000673  0.00029  NS  0.08714  0.00039  0.01520 |
